# Behavior Change Techniques in Physical Activity eHealth Interventions for People With Cardiovascular Disease: Systematic Review

**DOI:** 10.2196/jmir.7782

**Published:** 2017-08-02

**Authors:** Orlaith Mairead Duff, Deirdre MJ Walsh, Bróna A Furlong, Noel E O'Connor, Kieran A Moran, Catherine B Woods

**Affiliations:** ^1^ MedEx Wellness School of Health and Human Performance and Insight Centre for Data Analytics Dublin City University Dublin Ireland; ^2^ Insight Centre for Data Analytics Dublin City University Dublin Ireland; ^3^ School of Health and Human Performance and Insight Centre for Data Analytics Dublin City University Dublin Ireland; ^4^ Department of Physical Education and Sport Sciences Faculty of Education and Health Sciences University of Limerick Limerick Ireland

**Keywords:** systematic review, exercise, behavior, telemedicine, cardiovascular disease

## Abstract

**Background:**

Cardiovascular disease (CVD) is the leading cause of premature death and disability in Europe, accounting for 4 million deaths per year and costing the European Union economy almost €196 billion annually. There is strong evidence to suggest that exercise-based secondary rehabilitation programs can decrease the mortality risk and improve health among patients with CVD. Theory-informed use of behavior change techniques (BCTs) is important in the design of cardiac rehabilitation programs aimed at changing cardiovascular risk factors. Electronic health (eHealth) is the use of information and communication technologies (ICTs) for health. This emerging area of health care has the ability to enhance self-management of chronic disease by making health care more accessible, affordable, and available to the public. However, evidence-based information on the use of BCTs in eHealth interventions is limited, and particularly so, for individuals living with CVD.

**Objective:**

The aim of this systematic review was to assess the application of BCTs in eHealth interventions designed to increase physical activity (PA) in CVD populations.

**Methods:**

A total of 7 electronic databases, including EBSCOhost (MEDLINE, PsycINFO, Academic Search Complete, SPORTDiscus with Full Text, and CINAHL Complete), Scopus, and Web of Science (Core Collection) were searched. Two authors independently reviewed references using the software package Covidence (Veritas Health Innovation). The reviewers met to resolve any discrepancies, with a third independent reviewer acting as an arbitrator when required. Following this, data were extracted from the papers that met the inclusion criteria. Bias assessment of the studies was carried out using the Cochrane Collaboration’s tool for assessing the risk of bias within Covidence; this was followed by a narrative synthesis.

**Results:**

Out of the 987 studies that were identified, 14 were included in the review. An additional 9 studies were added following a hand search of review paper references. The average number of BCTs used across the 23 studies was 7.2 (range 1-19). The top three most frequently used BCTs included information about health consequences (78%, 18/23), goal setting (behavior; 74%, 17/23), and joint third, self-monitoring of behavior and social support (practical) were included in 11 studies (48%, 11/23) each.

**Conclusions:**

This systematic review is the first to investigate the use of BCTs in PA eHealth interventions specifically designed for people with CVD. This research will have clear implications for health care policy and research by outlining the BCTs used in eHealth interventions for chronic illnesses, in particular CVD, thereby providing clear foundations for further research and developments in the area.

## Introduction

Cardiovascular disease (CVD) is the leading cause of mortality worldwide, accounting for 30% of global deaths and 48% of deaths in Europe [1]. Cardiac rehabilitation (CR), which is used to reduce the impact of CVD and to promote healthy behaviors and active lifestyles for those with CVD [2], has been shown to improve physical health and decrease subsequent morbidity and mortality rates in CVD populations [3]. The main modality of CR is exercise. Two systematic reviews of exercise-based CR, which included 48 randomized controlled trials (RCTs), showed a 20% reduction in all-cause mortality and a 27% reduction in cardiac mortality at 2 to 5 years [4,5].

The efficacy of standard CR has been extensively reviewed. In terms of mortality rates, a systematic review and meta-analysis of 34 RCTs (n=6111 myocardial infarction patients) showed that those who attended CR had a lower risk of all-cause mortality than non-attendees (odds ratio=0.74, 95% CI 0.58-0.95) [6]. With respect to hospital admissions, a Cochrane review of 33 RCTs (n=4740 patients with heart failure) showed that CR reduced the risk of overall hospitalization (relative risk, RR=0.75, 95% CI 0.62-0.92; absolute risk reduction, ARR=7.1%; number needed to treat, NNT=15) and hospitalization for heart failure (RR=0.61, 95% CI 0.46-0.80; ARR=5.8%; NNT=18) [7]. A US observational study (n=635 coronary heart disease [CHD] patients) reported improvements in depression, anxiety, and hospital scores after CR [8]. CR has also been found to improve psychological well-being and to facilitate an improvement in quality of life. One of the most significant benefits of CR exercise training to participants is the improvement in aerobic capacity and cardiorespiratory fitness [9].

Even though CR has been shown to be effective, adherence to these programs is generally suboptimal. Participation rates in CR are documented at less than 50% worldwide [10]. Results from a Cochrane systematic review revealed that common barriers to adherence to CR programs included accessibility and parking at local hospitals, a dislike of group environments and work or domestic commitments [3]. In 2012, a *Heart* journal editorial concluded that CR should not only focus on content such as CHD risk factor modification and medication adherence but should also focus on the delivery mechanisms, thereby offering a range of different delivery methods for people according to their preferences and needs and potentially addressing the issue of low levels of participation [11]. The delivery of CR to date has largely been center-based, either in hospitals or community centers. However, in more recent times, there has been a shift toward a more home-based model of care. A systematic review by Dalal and colleagues [3] found that both home- and center-based forms of CR are equally effective in improving clinical and health-related quality of life outcomes in patients with CVD, suggesting the further provision of additional evidence-based home CR programs. A Cochrane review found that home-based interventions may be superior in terms of adherence to exercise, especially in the long term [12]. This would ensure that patients are given the choice of participating in a more traditional supervised center-based program or a home-based program, based on their personal preference.

The emerging area of electronic health (eHealth), defined as the use of information and communication technologies (ICTs) for health [13] may provide this alternative home-based delivery method. Interventions that encompass ICT (eg, Internet- and mobile-based communications and wearable monitors) enable the efficient delivery of educational resources, individually tailored health and wellness programs, as well as time-unlimited feedback, coaching and support [14]. Technology solutions for physical activity (PA) uptake and monitoring are being undertaken as a new mode of facilitating behavior change and may impact the current delivery of CR [15]. Telerehabilitation solutions refer to the use of ICT to provide rehabilitation services to people. Literature in this area for cardiac patients indicates that such interventions are feasible and effective when compared with conventional center-based CR [16].

Furthermore, eHealth interventions have been showing promising results in CR, supporting behavior change, clinical improvement, and improved social functioning. In 2013, Beatty and colleagues [17] conducted a review of mobile interventions for CR, identifying only three studies for inclusion. More recently, the interest in eHealth and mobile health (mHealth) has risen dramatically, indicating the increased focus in this field over recent years. Buys and colleagues [15] investigated the interest among cardiac patients in technology-enabled cardiovascular rehabilitation. Of the 298 patient (77% male; mean age 61.7 years [SD 14.5]) questionnaires included in the analysis, 97% had a mobile phone and 91% used the Internet. PA monitoring was reported by 12% of the respondents. Overall, cardiac patients showed a high interest in CR support through the Internet (77%) and mobile phones (68%). These findings suggest that patients with CVD show an interest in technology-enabled home-based CR, potentially allowing exercise-based rehabilitation programs to be more effective by making them more accessible, personalized, and more interactive with patients.

Behavior change techniques (BCTs) are integral to the design of complex health service interventions such as CR. A BCT is defined as “an observable, replicable, and irreducible component of an intervention designed to alter or redirect causal processes that regulate behavior, that is, a technique is proposed to be an ‘active ingredient’” [18]. The Medical Research Council (MRC) guidelines recommend the application of behavior change theory within complex health service interventions to allow for a theoretical understanding of behavior change [19]. The National Institute for Health and Care Excellence (NICE; [20]) guidelines on individual-level behavior change interventions aimed at changing health-damaging behaviors such as unhealthy diet, physical inactivity, excessive alcohol consumption, unsafe sex, and smoking, recommend the use of evidence-based BCTs, which have been proven to be effective at changing behavior such as goals and planning, feedback and monitoring, and social support. Despite this guidance, few interventions pay close attention to the behavior change theory and techniques used to design their interventions. In particular, the poor description of interventions in research protocols and published reports presents a barrier for future design of complex interventions [21], as it is difficult to identify the active and effective components of the intervention [18]. The proliferation of eHealth interventions requires the coding of such interventions to facilitate future research to compare accurately across interventions. With that in mind, this systematic review aims to identify the key BCTs applied in eHealth PA interventions for adults with CVD.

## Methods

This systematic review is reported in line with the Preferred Reporting Items for Systematic Reviews and Meta-Analyses (PRISMA) guidance. The inclusion criteria for studies were as follows: human- and quasi-RCTs, published and unpublished, of PA eHealth interventions for adults (aged ≥18 years) clinically diagnosed with CVD. Studies were included if the main intervention component was delivered via a computer, mobile phone, tablet, or phone (eg, mobile phone app, emails, text messages, and phone calls) with the primary or secondary aim of increasing the PA level of the user. The interventions could be delivered to groups or individuals. The inclusion criteria was kept quite broad to identify as many studies as possible with PA as a primary or secondary outcome, as well as studies that had PA as a component of the intervention.

The behavior change taxonomy version 1 was used to identify the specific BCTs used within the included studies [18]. Two researchers coded for the BCTs using the taxonomy.

### Outcome Measures

A description of the BCTs and their frequency of use in the 23 eHealth interventions reviewed were classified using a BCT taxonomy by Michie and colleagues. Due to the heterogeneous nature of the studies differing in PA outcome measures and time points, we were unable to carry out a meta-analysis examining the effectiveness of the BCTs in relation to the PA outcomes.

### Search Methods for the Identification of Studies

Seven electronic databases were searched, including MEDLINE (via EBSCOhost, 2000-2016), PsycINFO (via EBSCOhost, 2000-2016), Academic Search Complete (via EBSCOhost, 2000-2016), SPORTDiscus (via EBSCOhost, 2000-2016), CINAHL Complete (via EBSCOhost, 2000-2016), Scopus (2000-2016) and Web of Science (Core Collection; 2000-2016).

The search was restricted to studies published in English between 2000 and 2016. The search strategy used keywords relating to PA, eHealth interventions, CVD and adults, as well as appropriate synonyms. Boolean operators were used to expand, exclude, or join keywords in the search, using the terms “AND” and “OR.” In all the databases, the searches were limited to the fields of abstract and title only. The search strategy for all databases is illustrated in the Multimedia Appendix 1.

### Selection of Studies

**Figure 1:**
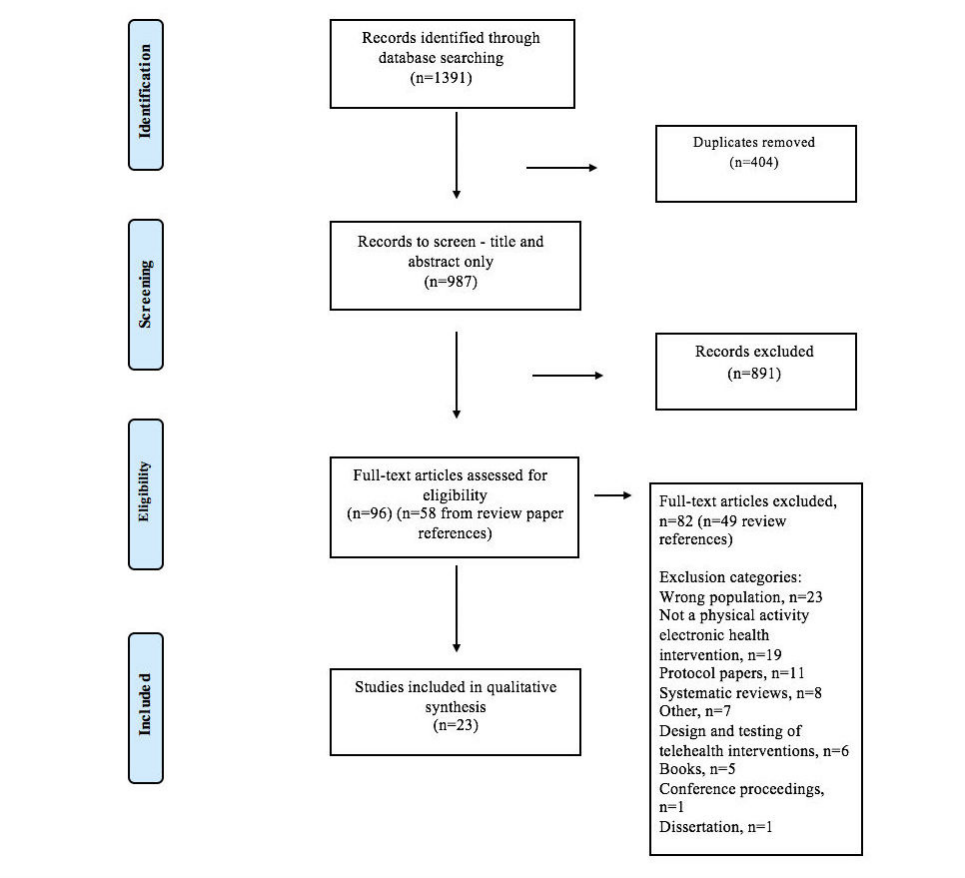
Preferred Reporting Items for Systematic Reviews and Meta-Analyses (PRISMA) flow diagram of reviewed and included studies.

Figure 1 shows the PRISMA flow diagram of reviewed and included studies. One researcher conducted the database search. All the studies identified following the database search were then uploaded to the Web-based systematic review software package “Covidence” (Veritas Health Innovation). First, a title and abstract review of all studies was completed independently by 2 authors. Any disagreements were discussed until a consensus was reached, or a third reviewer helped to resolve the discrepancy. A record was kept of all the studies excluded and the reason for exclusion via Covidence. Second, all the studies that met the inclusion criteria went through a full-text screening process by the 2 authors independently. Again, any disagreements between the authors on the eligibility of the studies were reviewed by a third author. Additional studies were also identified for inclusion by reviewing the reference lists of review papers through a hand search.

### Data Extraction

Data from the studies were extracted independently by 2 review authors using a data extraction template. Data extracted from the studies included study title, authors, country, year, patient group (sample size), inclusion criteria, study design, technology involvement, assessment, intervention details, outcomes, theory involved, BCTs identified, and results. No blinding to study author, institution, or journal occurred during the screening process for the study.

If multiple publications of the same study were identified, the team would try to extract and combine all the available data; where there was doubt, the original publication would be given priority. If data seemed to be missing from a study, we tried to obtain the missing data through correspondence with the study authors. The review team resolved any disagreements regarding study eligibility through group discussion.

### Assessment of Risk Bias

**Figure 2:**
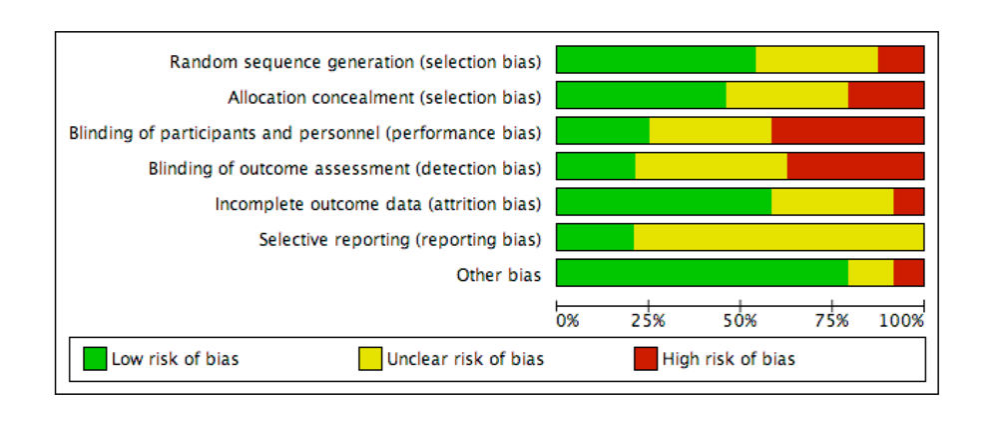
Risk of bias graph: review authors’ judgments about each risk of bias item presented as percentages across all included studies.

Two reviewers assessed each study for risk of bias (high, low, or unclear) using the Cochrane Collaboration’s risk of bias tool [22]. A third review author acted as arbitrator, if necessary. The results of the risk of bias assessment were then exported to RevMan to create a visual representation of the publication bias (see Figure 2).

### Assessing for Heterogeneity

Diversity across the studies was assessed qualitatively in terms of eHealth intervention, patient characteristics, and outcome measures.

### Data Synthesis

Following the extraction of data from the studies, careful consideration was given to the appropriateness of conducting a meta-analysis. As the studies were too heterogeneous to combine statistically, the data were synthesized qualitatively.

### Behavior Change Techniques

To gain an understanding of the types of BCTs used in PA eHealth interventions in this patient population, 2 authors screened the included studies and coded the BCTs used in each study using Michie and colleagues BCT taxonomy [18].

## Results

The search criteria returned 1391 studies through the database search. A total of 404 duplicates were removed, leaving 987 studies to screen. The title and abstracts of the studies were then screened by 2 reviewers, resulting in 891 records excluded for not meeting the inclusion criteria. The authors reviewed the full text of 96 studies, identifying 14 studies for inclusion in this review. From a hand search of review paper references, an additional 58 studies were identified as potentially eligible. Following a full-text review of these papers, nine studies were included in the review. Therefore, a total of 23 studies were included in the qualitative synthesis.

### Study Characteristics

For an overview of the included studies and the PA results, see Multimedia Appendix 2. Of the 23 studies included, 14 studies comprised an Internet or Web-based program and/or mobile phone intervention [23-36], three studies were telephone interventions [37-39], two studies used a telehealth device [40,41], and the remaining two studies comprised a form of telemonitoring [42,43]. Single studies comprising videoconferencing [44] and virtual reality wraparound screens [45] were also found. Of the 20 studies with a control group, 17 involved “usual care” as the control. Usual care predominately pertained to receiving standard CR services [25-29,31,33-37,39-42,44,45]. Eight studies were carried out in Europe [23,24,27,28,30,32,39,43], whereas seven of the studies were conducted in North America/South America [29,32,34,36,37,40,41]. Three studies apiece were conducted in Australia [25,35,38] and New Zealand [26,31,44], and two studies were conducted in Asia [25,42].

The majority of participants were recruited from hospitals/medical centers [23-27,29,30,32-41,43,45]. One study recruited participants from a general practitioner CHD registry [30], whereas another recruited from a CR referral list [38]. Tomita and colleagues [34] recruited participants from 3 hospitals and 2 health insurance companies. One study recruited participants from primary and community health services [35]. Outcomes were assessed from 3 weeks [41] to 16 months [39], with the average end point across the 23 studies at 4.5 months.

### Behavior Change Techniques

Only two out of the 23 studies explicitly mentioned the BCTs applied [26,27]. From the other studies, 2 reviewers coded the BCTs from the program description. Multimedia Appendix 3 outlines the number of BCTs used in each study as well a comprehensive list of the techniques used. The average number of BCTs employed in the included studies was 7.2 (range: 1-14). The top three most frequently used BCTs were information about health consequences (78%; 18/23), goal setting (behavior; 74%, 17/23) and joint third, self-monitoring of behavior and social support (practical) (48%; 11/23 each) (see Multimedia Appendix 4). The Text4Heart study conducted by Dale and colleagues [26] employed 14 BCTs—using the maximum number of BCTs out of all the studies. These were goal setting (behavior), problem solving, review outcome goals, feedback on behavior, self-monitoring of behavior, social support (unspecified), instruction on how to perform the behavior, information about health consequences, demonstration of the behavior, social comparison, prompts/cues, graded tasks, credible source, and reduce negative emotions. A study by Barnason and colleagues [41] used the lowest number of BCTs of the 23 studies included in the review, employing just one BCT - graded tasks.

The most common BCT group used in the 23 included studies was feedback and monitoring, whereas the second most common group was goals and planning. This was followed by social support. Four groups that did not appear in any of the 23 included studies were identity, scheduled consequences, self-belief, and covert learning.

Multimedia Appendix 4 outlines the frequency of use of the BCTs across the 23 studies, the BCT taxonomy group, and an example of how a BCT was incorporated into a study. Only two BCTs were used in over 70% of the studies; these were 5.1 information about health consequences (78%) and 1.1 goal setting (behavior; 74%). Additionally, four BCTs were used in over 40% of the studies; these included 2.2 feedback on behavior (43%), 2.3 self-monitoring of behavior (48%), 3.2 social support (practical; 48%), and 4.1 instruction on how to perform the behavior (43%). Several BCTs, including 10.3 nonspecific reward, 12.1 restructuring the physical environment, 12.5 adding objects to the environment, 11.1 pharmacological support, 6.1 demonstration of the behavior, 6.2 social comparison, 1.7 review outcome goals, 10.4 social reward, and 1.8 behavioral contract were only used in one study (see Multimedia Appendix 4 for more details).

**Table 1:** Frequency of behavior change techniques (BCTs) used in the studies with improved physical activity (PA) outcome.

Behavior change technique label	Total number of studies N=8 n (%)
1.1 Goal setting (behavior)	6 (75)
5.1 Information about health consequences	6 (75)
2.2 Feedback on behavior	5 (63)
4.1 Instruction on how to perform the behavior	5 (63)
2.3 Self-monitoring of behavior	4 (50)
3.2 Social support (practical)	4 (50)
3.1 Social support (unspecified)	3 (38)
9.1 Credible source	3 (38)
1.2 Problem solving	2 (25)
1.5 Review behavior goals	2 (25)
3.3 Social support (emotional)	2 (25)
7.1 Prompts/cues	2 (25)
8.7 Graded tasks	2 (25)
11.2 Reduce negative emotions	2 (25)
1.4 Action planning	1 (13)
2.4 Self-monitoring of outcomes of behavior	1 (13)
2.6 Biofeedback	1 (13)
2.7 Feedback on outcomes of behavior	1 (13)
10.4 Social reward	1 (13)
11.1 Pharmacological support	1 (13)
1.3 Goal setting (outcome)	0 (0)
1.7 Review outcome goals	0 (0)
1.8 Behavioral contract	0 (0)
2.1 Monitoring of behavior by others without feedback	0 (0)
2.5 Monitoring of outcomes of behavior without feedback	0 (0)
6.1 Demonstration of the behavior	0 (0)
6.2 Social comparison	0 (0)
10.3 Nonspecific reward	0 (0)
12.1 Restructuring the physical environment	0 (0)
12.5 Adding objects to the environment	0 (0)

### Behavior Change Techniques Linked to Improved Physical Activity Outcomes

Eight of the 15 interventions that had PA as an outcome measure reported statistically significant improvements in PA between the experimental and control groups. Goal setting (behavior) and information about health consequences were the most frequently used BCTs across the eight studies (n=6 each). This was followed by feedback on behavior and instruction on how to perform the behavior, which were incorporated in five studies each. The following BCTs were also included in the interventions that had an improved PA outcome at the final end point: self-monitoring of behavior, social support (practical), social support (unspecified), credible source, problem solving, review behavior goals, social support (emotional), prompts/cues, graded tasks, reduce negative emotions, action planning, self-monitoring of outcomes of behavior, biofeedback, feedback on outcomes of behavior, social reward, and pharmacological support (Table 1).

It is worth noting that the interventions that did not demonstrate a significant increase in PA (n=5) were at par with the level achieved in standard CR, as no significant differences between the control and experimental groups were found. This is an important finding, as it highlights the fact that the eHealth interventions were at par with or were significantly better at improving PA levels of cardiac patients when compared with standard cardiac services. This emphasizes the potential of eHealth interventions in a CR setting.

To further examine the efficacy of the individual BCTs, the interventions were grouped into four groups depending on whether PA was measured objectively or subjectively and whether there was a difference between experimental and control groups. Once the interventions were grouped, we sought to examine whether there were any common BCTs used across the studies (see Multimedia Appendix 5). This task allowed us to examine whether there were any similarities between the interventions in terms of the BCTs they employed. Objective and self-report studies with no difference between experimental and control groups were the only groups with similarities in the BCTs they employed. Social support (practical) and information about health consequences were employed in all self-report studies where there was no PA difference between the experimental and control groups. Goal setting (behavior) and feedback on behavior were employed in all PA objectively measured interventions where no significant difference was found between groups at the final end point. However, there were no similarities in the BCTs used across all the effective interventions, regardless of whether PA was measured objectively or subjectively. Furthermore, the average number of BCTs used across significant interventions did not differ, as the studies that increased PA versus those that did not increase PA employed an average of seven BCTs.

## Discussion

### Summary

This systematic review comprised 23 studies reviewing the use of BCTs in PA eHealth interventions for adults with CVD. To our knowledge, this is the first review that aimed to identify the use of Michie and colleagues behavior change taxonomy in PA eHealth intervention studies among this population. The findings of the review indicate that on an average, 7.2 BCTs were employed in the 23 studies. Information about health consequences was the most frequently used technique, with 78% of the studies incorporating this technique into their intervention. This was followed closely by goal setting (behavior), which was used in 74% of the studies, with self-monitoring of behavior and social support (practical) each employed in 48% of the studies.

Although Michie and colleagues BCT taxonomy is comprised of 93 different techniques, the maximum number of techniques used in a single intervention was 14 [26]. These were goal setting (behavior), problem solving, review outcome goals, feedback on behavior, self-monitoring of behavior, social support (unspecified), instruction on how to perform the behavior, information about health consequences, demonstration of the behavior, social comparison, prompts/cues, graded tasks, credible source, and reduce negative emotions. The minimum number of techniques used in a study was one - graded tasks [41]. A failing of the studies included in this review was the poor description of the intervention components. Only two studies in the review specifically mentioned the BCTs incorporated in their interventions [26,27]. However, even though the paper by Devi and colleagues [27] listed the BCTs used, it failed to link the BCTs used to the intervention functions or components. In the study by Dale [26], the researchers provided only examples of text messages linked to BCTs. None of the studies gave a full account of the BCTs used in their studies and how these were linked to the intervention components. This finding is in line with previous research, where reviews of nearly 1000 behavior change outcome studies found that interventions were fully and accurately described in only 5% to 30% of experimental studies [46-49]. Overall, this lack of robust and detailed information on the intervention functions provides a significant barrier to better understand the effects and mechanisms of behavior change interventions and to inform the development of more effective interventions in the future [16].

Another key issue relating to the poor description of behavior change interventions is the inconsistent use of terminology. This variation in terminology used makes the coding of the techniques used even more difficult when reviewing behavior change interventions. For example, social support (unspecified) was coded for in 39% of the studies included in the review by the reviewers. Terminology varied across the studies where social support was coded; for example, one study used a social reinforcement network [24], another incorporated mentors into their intervention [35], whereas another study involved tutorials in their intervention [33]. The reviewers coded these examples as social support (unspecified); however, this BCT was not specifically mentioned in any of the studies. Therefore, there is a need to have consistent terminology and sufficient information on intervention components to allow for the replication of interventions that have been found to be effective. The lack of such information appears to be particularly problematic in behavioral interventions rather than in pharmacological ones [21]. In a workshop, 26 multidisciplinary researchers were presented with behavioral or pharmacological intervention protocols and were asked whether the protocol provided sufficient information so that the study could be replicated in a practice setting. The researchers were less confident that they could replicate the behavioral interventions compared with the pharmacological interventions (*t*=6.45, *P*<.001) and concluded they would need more information for the replication of behavioral interventions (*U*=35.5, *P*=.02; [50]).

This review provides new and important information regarding the use of BCTs in eHealth PA for adults with CVD, highlighting the frequent use of the following BCTs: information about health consequences, goal setting (behavior), self-monitoring of behavior and social support (practical). However, it is clear that more robust and comprehensive interventions are needed, which systematically and coherently detail the BCTs used in the interventions. Identifying the active ingredients of the interventions will enable researchers to examine the effectiveness of these key intervention components, ensuring that the most effective BCTs are used regarding eHealth PA interventions for adults with CVD.

### Strengths and Limitations

A major strength of this review was the authors’ attempt to identify all relevant studies by using a comprehensive search strategy and multiple databases. The authors’ also hand-searched review paper references to identify any additional studies that may have been relevant to the review. All the studies identified following the database search were then uploaded to the Web-based systematic review software package “Covidence” (Veritas Health Innovation). This allowed for a systematic and comprehensive approach to screening the studies and coding the reasons for exclusion. This software also enabled the screening for risk of bias in a simple and efficient way. From this, a visual representation of the publication bias was produced using RevMan.

A limitation of this review was the wide variability among the studies included, with study designs ranging from RCTs, to feasibility studies and pilot trials. However, it was necessary to include all the studies and not just RCTs to identify as many PA eHealth interventions as possible. There was also a lack of consistency in the measurement of PA across the studies, from subjective to objective assessments. The follow-up duration also varied significantly from 3 weeks to 16 months. This meant that it was impossible to pool the results in a meta-analysis.

Many studies measured the physical fitness of their participants, as opposed to their PA levels. Although all the interventions had a PA/exercise component to their eHealth intervention, some studies did not directly measure the PA level of the participants. We can therefore only infer from the studies that by increasing PA behavior, the physical fitness outcome improved. This inference of a causal relationship between PA and physical fitness is a limitation to these studies. Another limitation is the variety of methods used to measure PA, meaning that the comparison between studies is challenging and therefore determining the impact of specific BCTs is impossible.

### Implications for Research and Practice

This systematic review highlights the need for more robust and comprehensive eHealth PA interventions for adults with CVD. Although the most frequently used BCTs were identified, it is worth noting that the majority of studies did not specifically detail the active ingredients of their interventions. Further work is also needed to determine the most appropriate measurement of PA among this population so that interventions use the best subjective and/or objective measurements, thereby ensuring that comparisons can be easily drawn across studies. This review also highlights the importance of identifying the BCTs used within a study and their link to the intervention components to understand the ingredients that bring about the desired behavior change. It is only by identifying these mechanisms of change that we can understand why an intervention was found to be effective or not.

## Appendix

Multimedia Appendix 1 Keyword searches.

Multimedia Appendix 2 Information on included studies.

Multimedia Appendix 3 Behavior change techniques used in the included studies.

Multimedia Appendix 4 Frequency of behavior change techniques (BCTs) used in the included studies.

Multimedia Appendix 5 Link between Interventions and behavior change techniques.

## Abbreviations

ARR: absolute risk reduction
BCTs: behavior change techniques
CHD: coronary heart disease
CR: cardiac rehabilitation
CVD: cardiovascular disease
eHealth: electronic health
ICT: information and communication technology
mHealth: mobile health
MRC: Medical Research Council
NICE: National Institute for Health and Care Excellence
NNT: number needed to treat
PA: physical activity
PRISMA: Preferred Reporting Items for Systematic Reviews and Meta-Analyses
RCT: randomized controlled trial
RR: relative risk
SFI: Science Foundation Ireland
