# Supplementary material for: Behavior Change Techniques in Physical Activity eHealth Interventions for People With Cardiovascular Disease: Systematic Review
Source: J Med Internet Res. 2017 Aug 2;19(8):e281. doi: 10.2196/jmir.7782 (PMC5559649; doi:10.2196/jmir.7782)
Supplement: Multimedia Appendix 1 [file jmir_v19i8e281_app1.pdf]

Multimedia Appendix 1 – Keyword Searches

Title: Systematic review of the use of behaviour change techniques (BCT's) in eHealth interventions for adults with cardiovascular disease.

Databases:

Limited to Published date: 2000-2016

Searched: 10.02.16

Ebscohost - MEDLINE, PsycINFO, Academic Search Complete, SPORTDiscus with Full Text, CINAHL Complete

Search No.	Search Terms	Results
S5	S1 AND S2 AND S3 AND S4	649
S4	TI ("physical activity" or "activity level" or "sedentary behaviour" or "sedentary behavior" or exercise or walking or running or recreation or "fun and games" or sport or pastime) OR AB ("physical activity" or "activity level" or "sedentary behaviour" or "sedentary behavior" or exercise or walking or running or recreation or "fun and games" or sport or pastime)	1,034,549
S3	TI (intervention or education or information or train or coach or skills or program* or counsel or mentor or support or "health promotion" or lifestyle or advocacy or communicate or empower or therapy or intensive or manage or psycho* or treat or rehabilitation or "disease management" or "disease treatment") OR AB (intervention or education or information or train or coach or skills or program* or counsel or mentor or support or "health promotion" or lifestyle or advocacy or communicate or empower or therapy or intensive or manage or psycho* or treat or rehabilitation or "disease management" or "disease treatment")	9,732,085
S2	TI ("coronary heart disease" or chd or "coronary artery disease" or cvd or cardiovascular) OR AB ("coronary heart disease" or chd or "coronary artery disease" or cvd or cardiovascular)	552,613
S1	TI ("mobile phone" or "cell phone" or "cellular phone" or smartphone or "smart phone" or "mobile technology" or telemedicine or mHealth or eHealth or wireless or "personal digital assistant" or computer or technology or digital) OR AB ("mobile phone" or "cell phone" or "cellular phone" or smartphone or "smart phone" or "mobile technology" or telemedicine or mHealth or eHealth or wireless or "personal digital assistant" or computer or technology or digital)	1,516,069

Scopus

**Search No.**	**Search Terms**	**Results**
S5	S1 AND S2 AND S3 AND S4	85
S4	TITLE-ABS("physical activity" or "activity level" of "sedentary behaviour" or "sedentary behavior" or exercise or walking or running or recreation or "fun and games" or sport or pastime)	28,263
S3	TITLE-ABS (intervention or education or information or train or coach or skills or program* or counsel or mentor or support or “health promotion” or lifestyle or advocacy or communicate or empower or therapy or intensive or manage or psycho* or treat or rehabilitation or “disease management” or “disease treatment”)	7,172,380
S2	TITLE-ABS ("coronary heart disease" or chd or “coronary artery disease" or cvd or cardiovascular)	371,781
S1	TITLE-ABS ("mobile phone" or "cell phone" or "cellular phone" or smartphone or "smart phone" or "mobile technology" or telemedicine or mHealth or eHealth or wireless or "personal digital assistant" or computer or technology or digital)	2,481,098

Web of Science (Core Collection)

**Search No.**	**Search Terms**	**Results**
S5	S1 AND S2 AND S3 AND S4	784
S4	TS=("physical activity" or "activity level" or "sedentary behaviour" or "sedentary behavior" or exercise or walking or running or recreation or "fun and games" or sport or pastime)	710,150
S3	TS=(intervention or education or information or train or coach or skills or program* or counsel or mentor or support or “health promotion” or lifestyle or advocacy or communicate or empower or therapy or intensive or manage or psycho* or treat or rehabilitation or “disease management” or “disease treatment”)	6,878,918
S2	TS=("coronary heart disease" or chd or “coronary artery disease" or cvd or cardiovascular)	422,096
S1	TS=("mobile phone" or "cell phone" or "cellular phone" or smartphone or "smart phone" or "mobile technology" or telemedicine or mHealth or eHealth or wireless or "personal digital assistant" or computer or technology or digital)	1,668,653

Note: TS= Title, Abstract, Author Keywords and Keywords Plus®
