# Supplementary material for: Behavior Change Techniques in Physical Activity eHealth Interventions for People With Cardiovascular Disease: Systematic Review
Source: J Med Internet Res. 2017 Aug 2;19(8):e281. doi: 10.2196/jmir.7782 (PMC5559649; doi:10.2196/jmir.7782)
Supplement: Multimedia Appendix 2 [file jmir_v19i8e281_app2.pdf]

Multimedia Appendix 2: Information on included studies

Author and country	Sample (n) (% males) / Dropout (% DO)	Intervention	Control	PA Measure and Effect
**Ades et al. 2000**<br><br>**USA**	133 (81.2% male); n=83 in the transtelephonically monitored rehab program and n=50 in the standard on-site rehab program<br><br>9% DO	Multiple long-distance phone lines to monitor up to 8 patients at the same time. Patients were given a patient's kit that included bipolar ECG leads, an ECG transmitter unit, a headset, a voice transmitter and a telephone modem. During the exercise sessions, patients were in direct telephone contact with the nurse coordinator and with other participants.	Standard on-site rehabilitation program	No physical activity measurement*
**Ammenwerth et al. 2015**<br><br>**Austria**	25 (96% male)<br><br>0 DO	Tele-monitoring programme is a multi-modal intervention programme to improve lifestyle and medication management of patients with CHD. It includes patient education, self-monitoring with goal-setting and feedback, and regular clinical visits.	No control group	Pedometer (steps)<br><br>Pre-defined goals for PA were reached in up to 86% ad 73% of days, respectively.
**Antypas et al. 2014**<br><br>**Norway**	69; n=29 tailored group (76% male) and n= 40 control group (79% male)<br><br>27.5% DO	Internet- based intervention-consisting of general information about CVD and self-management, including diet, physical activity, smoking and medication and a discussion forum, augmented by tailored messages.	Received the basic internet intervention. Did not receive messages or feedback.	IPAQ (MET minutes/week)<br><br>To = C > E, PA, (p=.02)<br><br>T1m = No PA diff, (p=.38)<br><br>T3m = E > C, PA, (p=0.02)
**Artinian et al. 2003**<br><br>**USA**	18 (94% male); n=9 in the intervention group and n=9 in the control group<br><br>0 DO	The Med- eMonitor retains medications in individual compartments, and uses an alarm to remind patients daily when to take their medications, which to take, and how many to take. The monitor contained an additional 25 virtual compartments in which daily reminders or questions about other medications, symptoms, monitoring daily weight and blood pressure, reducing salt intake, eating heart healthy, and engaging in physical activity. The monitor sat in a cradle that was connected to a telephone line - patient information was automatically transmitted to the Med-eMonitor server.	Received usual care	No physical activity measurement*

**Barnason et al. 2009**<br><br>**USA**	280; n=143 in the intervention group and n=137 in the control group<br><br>17.1% DO	Tele-health-intervention delivered via the Health Buddy ® telehealth device; for a total of 42 daily sessions. The symptom management intervention provided subjects with strategies designed to address commonly occurring symptoms experienced after recovery from CABS, to improve outcomes (physical activity, functioning); and a long-term outcome of having less healthcare utilization.	Received usual care	Accelerometer, modified 7-day activity interview (baseline PA assessment), physical activity and exercise diary<br><br>T6m = No sig. diff between exp and control groups in average daily activity counts (p=.20)
**Chow et al. 2015**<br><br>**Australia**	710; n=352 received text message intervention and n=358 received usual care<br><br>1.5% DO	A text message–based prevention program which involved delivery of regular semipersonalized text messages providing advice, motivation, and information that aimed to improve diet, increase physical activity, and encourage smoking cessation (if relevant).	Received usual care - includes community follow-up with the majority referred to inpatient cardiac rehabilitation by their physician	Global physical activity questionnaire (Total PA – MET minutes/week)<br><br>T6m = E > C, PA (p=.003)
**Chuang et al. 2006**<br><br>**Taiwan**	24 (100% male); n= 12 received non-virtual reality (VR) intervention and n=12 received VR intervention<br><br>16.7% DO	Intervention consisted of virtual reality "wraparound" screens used in CR.	No virtual reality experience during rehabilitation	No physical activity measurement*
**Dale et al. 2015**<br><br>**New Zealand**	123(81.3% male); n=61 in the intervention group and n=62 in the control group<br><br>9.8% DO	Theoretically framed comprehensive program of evidence-based CR guidelines delivered by text message and a supporting website over 24 weeks. Participants received 7 messages per week and had access to a supporting website. Participants were also given a pedometer to self-monitor their physical activity.	Received standard CR services	Godin leisure time physical activity questionnaire<br><br>Exp. group ↑ adherence to health behaviour from baseline (33%) to 3 months (59%) and plateaued at 6 months (53%).<br><br>Control group ↑ adherence to health behaviour from baseline (27%) to 3 months (37%) and plateaued at 6 months (39%).<br><br>Sig. treatment effect in favor of intervention at 3 months (p=.03) but not 6 months (p=.13)

**Dalleck et al. 2010**<br><br>**New Zealand**	226; n=173 conventional cardiac rehab (58% male) and n=53 in tele-medicine – delivered cardiac rehab (55% male)<br><br>0 DO	In the telemedicine site, rehabilitation was delivered via telemedicine using videoconferencing.	Conventional cardiac rehabilitation	No physical activity measurement*
**Devi et al. 2014**<br><br>**UK**	94 (74.5% male); n= 48 in the intervention group and n=46 in the usual care group<br><br>23.4% DO	6-week web-based rehabilitation program contained information about the secondary prevention of coronary heart disease (CHD) and set each user goals around physical activity, diet, managing emotions, and smoking. Participants completed an online exercise diary and communicated with rehabilitation specialists through an email link/synchronized chat room.	Received usual care from their GP	<u>Accelerometer (daily steps)</u><br><br>T6w = E > C, step count, (p=.02)
**Frederix et al. 2015**<br><br>**Belgium**	140; n=70 intervention group (96% male) and n=70 in control group (79%male)<br><br>10% DO	Internet-based telerehabilitation program as well as to conventional centre-based CR. Wore an accelerometer for the duration of the study - uploaded the data at least every 2 weeks to a secure webpage - patients could self-monitor PA via the webpage. Based on the uploaded data a semi-automatic tele-coaching system provided patients with feedback via email and/or SMS. Received dietary and smoking cessation tele-coaching programme.	Conventional cardiac rehab care	<u>Accelerometer (step data) and IPAQ (MET-min/week – Vigorous and/or moderate and/or walking (VMW))</u><br><br>T6w and T6m = Exp. Group, No sig. PA change (p=.24)<br><br>T6w and T6m = Control Group, No sig. PA change (p=.85)<br><br>T6m = E>C, leisure VMW, (p=.01)
**Furber et al. 2010**<br><br>**Australia**	215; n=104 in the intervention group (71.2% male) and n=111 in the control group (68.5% male)<br><br>6.5% DO	The intervention comprised a pedometer, a step calendar for self-monitoring, and telephone support which included goal setting and behavioural reinforcement. Goal setting varied from people nominating specific step count targets or a time-based target. Interactions with participants were carried out by mail and telephone.	Sent two PA information brochures by mail after they had completed the baseline questionnaire. The control group participants did not receive any reinforcement telephone calls	<u>Active Australia Questionnaire (Total PA and Walking)</u><br><br>T6w = E > C, total PA mins (p=0.027)<br>E > C, total PA sessions (p=0.003)<br>E > C, walking minutes (p=0.013)<br>E > C, walking sessions (p=0.002)<br><br>T6m = E > C, total PA mins (p=0.015)<br>E > C, total PA sessions (p=0.019)<br>E > C, walking minutes (p=0.002)<br>E > C, walking sessions (p=0.0026)

**Hanssen et al. 2007**<br><br>**Norway**	288; n= 156 in the intervention group (84.6% male) and n= 132 in the control group (76.5% male)<br><br>26% DO	All patients randomized to the intervention group received, in addition to the current clinical practice a structured intervention encompassing telephone follow-up and an open telephone line. The purpose of these calls was to provide patients with information, education, and support based on individual needs and to provide patients with information.	Received current clinical practice care -> one visit to a physician at the outpatient clinic 6-8 weeks after discharge and subsequent visits to the patient's general practitioner	<u>Single item question – How often on an average do you exercise each week? Scale answers.</u><br><br>T3m = No sig. diff between exp. and control groups in weekly exercise habits<br><br>T6m = E > C, PA (p=0.004)
**Lear et al. 2014**<br><br>**Canada**	78; n=38 in the intervention group (90% male) and n=40 in the control group (80% male)<br><br>9% DO	Virtual cardiac rehabilitation programme (vCRP). Participants were also provided with a heart rate and a home blood pressure monitor for the intervention. A webpage displayed the tasks that needed to be completed for each week. The one-on-one chat sessions were used to discuss progress, any change in symptoms, provide exercise prescription, dietary recommendations, and risk factor management. These sessions lasted ≈1 hour; however, participants could also access the vCRP health staff via email.	Received usual care	<u>Minnesota Leisure Time Physical Activity Questionnaire</u><br><br>T16m = No sig. diff between exp. group and control group in leisure time PA (p=0.191)
**Lee et al. 2013**<br><br>**Korea**	60; of the 55 participants who completed the follow-up 85% male in the intervention group and 76% male in the UC group.<br><br>8.3% DO	Intervention group received standard medical therapy and a CR programme consisted of educational rehabilitation and exercise training. Participants wore wireless monitoring equipment (HeartCall) to check their heart rate through electrocardiography. A phone call was made to participants once a week for counselling and to minimize risk factors and establish exercise intensity in stages	Usual care	No physical activity measurement*
**Lindsay et al. 2009**<br><br>**UK**	108 (66% male): n=58 intervention group and n=54 in the control group<br><br>0 DO	Internet health portal accessed via a computer and broadband. Access to project website and could interact in one of five closed groups, with facilitation from the researchers for the first 6 months. The website contained a glossary and information resources about CHD, diet, exercise and smoking. The moderators began discussion topics during the moderated phase. During the moderated phase, there were two forms of communication with moderators: discussion forms and instant messaging. There was an unmoderated phase for 3 months following the 6-month moderated phase.	Received a computer and 1-year broadband subscription like the experimental group however they did not have access to the project portal. Weekly drop-in session and phone support were available.	<u>Exercise frequency measured using a question from the Health Survey for England (days per week moderate exercise: mean + SD)</u><br><br>T9m = No sign. diff between exp. and control groups in days per week moderate exercise, (p=.703)

**Maddison et al. 2014**<br><br>**New Zealand**	171; n=85 intervention group (81% male) and n=86 control group (81% male)<br><br>10.5% DO	The HEART (Heart Exercise And Remote Technologies) intervention involved personalised automated package of text messages and a secure website with videos aimed at increasing exercise behaviour over a 24-week period. The intervention group also receive usual CR care.	Directed to receive usual community-based CR	IPAQ (minutes/week): Total PA, leisure time PA (LTPA) and walking<br><br>T6m = E > C, LTPA (p=0.04) and walking (p=.02)<br><br>T6m = No Total PA diff between group, (p=0.22)
**Piotrowicz et al. 2014**<br><br>**Poland**	365 (84% male)<br><br>0 DO	4-week home based cardiac telerehabilitation (HTCR) programme based on walking, Nordic walking or cycloergometer training. HTCR was tele-monitored with a device adjusted to register electrocardiogram (ECG) recording and to transmit data via mobile phone to the monitoring center.	No control group	No physical activity measurement*
**Reid et al. 2011**<br><br>**Canada**	223 (84.3% male); n=115 in the intervention group and n=108 in the control group<br><br>30.9% DO	CardioFit is an internet-based expert system and website. Participants received hospital visits from an exercise specialist who delivered a CardioFit personally tailored physical activity plan and instructions on how to access the CardioFit website. Following discharge, participants were asked to log their daily activity on the website and complete a series of five online tutorials. After each tutorial, a new PA plan was developed. Participants received motivational feedback on progress via email.	Usual care intervention: Received PA guidance from their attending cardiologist and an education booklet	Pedometer (steps/day) and Godin leisure time physical activity questionnaire (MVPA min/week)<br><br>T6m and T12m = E > C, Objective PA, (p=0.023)<br><br>T6m and T12m = E > C, Subjective PA, (p=0.047)
**Scalvini et al. 2009**<br><br>**Italy**	47 (87% male)<br><br>0 DO	One-month home rehabilitation programme supervised by a nurse-tutor and physiotherapist. Physiotherapy was performed at home with callisthenic exercises and bicycle-ergometer tests. Tele-monitoring and scheduled contacts with a nurse. Tele-assistance if required.	No control group	No physical activity measurement*
**Tomita et al. 2009**<br><br>**USA**	40 (32.5% male); n= 16 in the intervention group and n=24 in the control group<br><br>20% DO	Publicly accessible and secured websites. Informational support included online information on: HF, drugs used to treat HF, effects of alcohol and smoking, depression, prescribed home exercise, nutrition, weight management, and exercise. An exercise instruction program was developed and delivered via streaming video. Participants were asked to access the website daily to record their vital signs and health behaviors. Health care professionals emailed participants feedback for patients' records.	Received usual care	Knowledge questionnaire developed for the study – Exercise frequency question as part of questionnaire<br><br>T12m = E > C, PA (p=0.001)
**Varnfield et al. 2014**<br><br>**Australia**	120; n=60 in intervention and n=60 in control group<br><br>36.6% DO	The CAP-CR platform used a smartphone for health (health diary) and exercise monitoring (inbuilt accelerometer). Through the smartphone participants received motivational and educational materials via text messages and there were also preinstalled audio and	Traditional centre-based CR	No physical activity measurement*

		video files (e.g. understanding CVD, symptoms and managements)		
**Widmer et al. 2015**<br><br>**USA**	76; n=25 in CR+PHA group (76% male), n=19 in CR group (89% male), n=17 in Post-CR+PHA group (65% male) and n=15 in post-CR group (53% male)<br><br>0 DO	Intervention groups used an online and smartphone version of a personal health assistant (PHA) during CR and Post-CR.	Two control groups: Usual Mayo clinic CR group and usual post-CR care group	<u>Input PA minutes per day into personal health assistant.</u><br><br>T3m = Exp. Group ↑ PA (p= <0.0001)<br>Control. Group ↑ PA (p= <0.0001)<br><br>T3m = No PA diff between groups (p=.24)
