# Supplementary material for: Behavior Change Techniques in Physical Activity eHealth Interventions for People With Cardiovascular Disease: Systematic Review
Source: J Med Internet Res. 2017 Aug 2;19(8):e281. doi: 10.2196/jmir.7782 (PMC5559649; doi:10.2196/jmir.7782)
Supplement: Multimedia Appendix 3 [file jmir_v19i8e281_app3.pdf]

Multimedia Appendix 3: Behaviour change techniques used in the included studies

	Behaviour Change Technique Codes																														
**Study**	**1.1**	**1.2**	**1.3**	**1.4**	**1.5**	**1.6**	**1.7**	**1.8**	**2.1**	**2.2**	**2.3**	**2.4**	**2.5**	**2.6**	**2.7**	**3.1**	**3.2**	**3.3**	**4.1**	**5.1**	**6.1**	**6.2**	**7.1**	**8.7**	**9.1**	**10.3**	**10.4**	**11.1**	**11.2**	**12.1**	**12.5**
**Ades et al. (2000)**	✓			✓						✓				✓			✓			✓					✓						
**Ammenworth et al. (2015)**	✓		✓						✓	✓	✓	✓	✓	✓	✓	✓	✓			✓											
**Antypas et al. (2014)**	✓			✓						✓						✓			✓	✓			✓								
**Artinian et al. (2003)**	✓			✓				✓			✓	✓		✓	✓					✓			✓		✓						
**Barnason et al. (2009)**																								✓							
**Chow et al. 2015**		✓															✓			✓			✓		✓						
**Chuang et al. (2006)**			✓								✓		✓	✓																✓	✓
**Dale et al. (2015)**	✓	✓					✓			✓	✓					✓			✓	✓	✓	✓	✓	✓	✓				✓		
**Dalleck et al. 2010**	✓			✓	✓								✓						✓												

	Behaviour Change Technique Codes																														
**Study**	1.1	1.2	1.3	1.4	1.5	1.6	1.7	1.8	2.1	2.2	2.3	2.4	2.5	2.6	2.7	3.1	3.2	3.3	4.1	5.1	6.1	6.2	7.1	8.7	9.1	10.3	10.4	11.1	11.2	12.1	12.5
**Devi et al. 2014**	✓				✓					✓	✓									✓				✓			✓		✓		
**Frederix et al. (2015)**	✓									✓							✓		✓	✓				✓				✓			
**Furber et al. (2010)**	✓	✓			✓					✓	✓			✓		✓															
**Hanssen et al. (2007)**	✓																	✓	✓	✓					✓						
**Lear et al. (2014)**	✓			✓									✓	✓			✓		✓	✓											
**Lee et al. (2013)**	✓											✓		✓						✓				✓							
**Lindsay et al. (2009)**																✓	✓			✓											
**Maddison et al. (2014)**	✓	✓		✓							✓						✓			✓					✓						

	Behaviour Change Technique Codes																														
**Study**	**1.1**	**1.2**	**1.3**	**1.4**	**1.5**	**1.6**	**1.7**	**1.8**	**2.1**	**2.2**	**2.3**	**2.4**	**2.5**	**2.6**	**2.7**	**3.1**	**3.2**	**3.3**	**4.1**	**5.1**	**6.1**	**6.2**	**7.1**	**8.7**	**9.1**	**10.3**	**10.4**	**11.1**	**11.2**	**12.1**	**12.5**
**Piotrowicz et al. (2014)**	✓											✓	✓	✓					✓	✓											
**Reid et al. (2011**	✓									✓	✓					✓	✓		✓												
**Scalvini et al. (2009)**	✓			✓					✓				✓			✓			✓	✓					✓						
**Tomita et al. (2009)**											✓	✓			✓	✓	✓	✓	✓	✓					✓				✓		
**Varnfield et al. (2014)**	✓									✓	✓	✓		✓	✓	✓	✓			✓									✓		
**Widmer et al. (2015)**			✓							✓	✓	✓			✓	✓	✓			✓			✓	✓		✓					

Note: The behaviour change technique codes listed in table 2 are the BCTs identified in the 23 studies included in the review. These BCTs are part of the Michie's taxonomy of 93 consensually agreed, distinct BCTs. The following are each of the code names; 1.1 Goal setting (behaviour), 1.2 Problem solving, 1.3 Goal setting (outcome), 1.4 Action planning, 1.5 Review behaviour goal(s), 1.6 Discrepancy between current behaviour and goal, 1.7 Review outcome goals, 1.8 Behavioural contract, 2.1 Monitoring of behaviour by others without feedback, 2.2 feedback on performance, 2.3 Self-monitoring of behaviour, 2.4 Self-monitoring of outcome(s) of behaviour, 2.5 Monitoring of outcome(s) of behaviour without feedback, 2.6 Biofeedback, 2.7 Feedback on outcome(s) of behaviour, 3.1 Social support (unspecified), 3.2 Social support (practical), 3.3 Social support (emotional), 4.1 Instruction on how to perform the behaviour , 5.1 information about health consequences, 6.1Deomstration of the behaviour, 6.2 Social comparison, 7.1 Prompts/cues, 8.7 Graded tasks, 9.1 Credible source, 10.3 Non-specific reward, 10.4 Social reward, 11.1pharmacological support, 11.2 Reduce negative emotions, 12.1Restructuring the physical environment, 12.5 Adding objects to the environment.
