# Supplementary material for: Behavior Change Techniques in Physical Activity eHealth Interventions for People With Cardiovascular Disease: Systematic Review
Source: J Med Internet Res. 2017 Aug 2;19(8):e281. doi: 10.2196/jmir.7782 (PMC5559649; doi:10.2196/jmir.7782)
Supplement: Multimedia Appendix 4 [file jmir_v19i8e281_app4.pdf]

Multimedia Appendix 4: Frequency of BCTs used in the included studies

BCT label	BCT group	Example of how the BCT was used	Frequency of use (%)	Studies where found
**5.1 Information about health consequences**	Natural consequences	*"…The website contained a glossary and information resources about CHD, diet, exercise and smoking…"* [16]	18 (78%)	1-4, 6, 8, 10-11, 13-18, 20-23
**1.1 Goal setting (behaviour)**	Goals and planning	*"…Together with a MyCor physician, individuals goal for blood pressure, footsteps and weight were defined…"* [2]	17 (74%)	1-4, 8-15, 17-20, 22
**2.3 Self-monitoring of behaviour**	Feedback and monitoring	*"…Following hospital discharge, participants were asked to log their daily activity on the CardioFit website…"* [19]	11 (48%)	2, 4, 7-8, 10, 12, 17, 19, 21-23
**3.2 Social support (practical)**	Social support	*"…All participants received detailed programme information and 1h of face-to-face training on technology use…"* [22]	11 (48%)	1-2, 6, 11, 14, 16-17, 19, 21-23
**4.1 Instruction on how to perform the behaviour**	Shaping knowledge	*"… Tutorials were organised to engage self-control processes including exercise planning, goal-setting, monitoring and self-regulation, and relapse prevention…"* [19]	10 (43%)	3, 8-9, 11, 13-14, 18-21
**2.2 Feedback on behaviour**	Feedback and monitoring	*"… patients were provided an individual, automatic feedback report that was sent to their smartphone once weekly…"* [2]	10 (43%)	1-3, 8, 10-12, 19, 22-23
**2.6 Biofeedback**	Feedback and monitoring	*"…In general, these patients were monitored electrocardiographically for their first 4 to 6 sessions with exercise intensity guided by a progressive heart rate prescription of 65% to 85% of their maximal measured heart rate from the baseline stress test…"* [1]	9 (39%)	1, 2, 4, 7, 12, 14-15, 18, 22
**3.1 Social support (unspecified)**	Social support	*"…Social reinforcement network that encourages the adoption and maintenance of a healthier lifestyle for improved wellness…"* [23]	9 (39%)	3, 8, 12, 16, 19-23
**9.1 Credible source**	Comparison of outcomes	*"…Role model video vignettes…"* [17]	8 (35%)	1, 4, 6, 8, 13, 17, 20-21
**2.4 Self-monitoring of outcomes of**	Feedback and monitoring	*"…Each participant was equipped with a smartphone preinstalled with health diary and activity monitoring*	7 (30%)	2, 4, 15, 18, 21-23

**behaviour**		*applications; blood pressure monitor and weight scale…"* [22]		
**1.4 Action planning**	Goals and planning	*"…The home programme consisted of regular exercise prescription…"* [17]	7 (30%)	1, 3-4, 9, 14, 17, 20
**8.7 Graded tasks**	Repetition and substitution	*"…Exercise intensity was 40% weeks 2 to 4, 50% weeks 5 to 6, 60% weeks 7 to 8, 70% weeks 9 to 10, and 80% weeks 11 to 12…"* [15]	6 (26%)	5, 8, 10-11, 15, 23
**2.7 Feedback on outcome(s) of behaviour**	Feedback and monitoring	*"…If patients confirmed these values, messages to consult their physician appeared, as these changes in weight, blood pressure, or lab values could represent a potential danger to their health…"* [23]	5 (22%)	2, 4, 21-23
**2.5 Monitoring of outcomes of behaviour without feedback**	Feedback and monitoring	*"…Participants were asked to wear their heart rate monitors when exercising and upload their exercise data at least twice per week to the vCRP…"* [14]	5 (22%)	3, 5, 7-8, 24
**1.2 Problem solving**	Goals and planning	*"…Participants were asked to develop strategies to overcome barriers to increasing their physical activity…"* [12]	4 (17%)	2, 6, 13, 23
**11.2 Reduce negative emotions**	Regulation	*"…Emotional support was provided on an as needed basis via email…"* [21]	4 (17%)	12-13, 16-17
**7.1 Prompts/cues**	Associations	*"… Have you gone for your walk today jane?…"* [6] *(message)*	3 (13.0%)	6, 8, 10
**1.5 Review behaviour goals**	Goals and planning	*"…Baseline and post-programme appointments between the registered dietician and patients at the telemedicine-supported site were conducted by telemedicine…"* [9]	3 (13%)	3, 16, 23
**1.3 Goal setting (outcome)**	Goals and planning	*"…The PHA provides user-friendly and interactive access to… targets, plans…"* [23]	3 (13%)	4-5, 7
**2.1 Monitoring of behaviour by others without feedback**	Feedback and monitoring	*"…In addition, video-recorders (cassette or DVD) for physiotherapy and 1-lead ECG devices were provided. An electronic health record was prepared for each patients and the patients general practitioner informed…"* [20]	2 (9%)	7, 19

**3.3 Social support (emotional)**	Social support	*"…Providing alternative coping strategies when the patient appeared to use inappropriate strategies…"* [13]	2 (9%)	17, 20
**10.3 Non-specific reward**	Reward and threat	*"…The PHA provides user-friendly and interactive access to… awards that encourages the adoption and maintenance of a healthier lifestyle for improved wellness…"* [23]	1 (4%)	4
**12.1 Restructuring the physical environment**	Antecedents	*"…The virtual terrain in our study consisted of a 5-km-long straight (or curved) stretch of road, grass and trees with a mountain background…"* [7]	1 (4%)	5
**12.5 Adding objects to the environment**	Antecedents	*"…The images were projected from behind the viewer through 3 projectors connected with computers…"* [7]	1 (4%)	5
**11.1 Pharmacological support**	Regulation	*"…The smoking cessation tele-coaching program included nicotine replacement therapy…"* [11]	1 (4%)	10
**6.1 Demonstration of the behaviour**	Comparison of behaviour	*"Video role model messages"* [8]	1 (4%)	13
**6.2 Social comparison**	Comparison of behaviour	*"Video role model messages"*[8]	1 (4%)	13
**1.7 Review outcome goals**	Goals and planning	*"…You're a few weeks into the program. Well done! Time to add a new goal - maybe you are ready to think about a long-term goal this time…"* [8] *(message)*	1 (4%)	13
**10.4 Social reward**	Reward and threat	*Paper listed the behaviour change techniques used in the intervention. Social reward was one of the BCT's used.*	1 (4%)	16
**1.8 Behavioural contract**	Goals and planning	*"…Provided patients with written information that included the name, frequency and action of their medications…"* [4]	1 (4%)	22
