# Supplementary material for: Behavior Change Techniques in Physical Activity eHealth Interventions for People With Cardiovascular Disease: Systematic Review
Source: J Med Internet Res. 2017 Aug 2;19(8):e281. doi: 10.2196/jmir.7782 (PMC5559649; doi:10.2196/jmir.7782)
Supplement: Multimedia Appendix 5 [file jmir_v19i8e281_app5.pdf]

Multimedia Appendix 5: Link between Interventions and Behaviour Change Techniques

Self-report studies with no difference between experimental and control groups

**Study**	**Behaviour Change Technique Codes**																														
	**1.1**	**1.2**	**1.3**	**1.4**	**1.5**	**1.6**	**1.7**	**1.8**	**2.1**	**2.2**	**2.3**	**2.4**	**2.5**	**2.6**	**2.7**	**3.1**	**3.2**	**3.3**	**4.1**	**5.1**	**6.1**	**6.2**	**7.1**	**8.7**	**9.1**	**10.3**	**10.4**	**11.1**	**11.2**	**12.1**	**12.5**
**Lear et al. (2014)**	✓			✓									✓	✓			✓		✓	✓											
**Lindsay et al. (2009)**																✓	✓			✓											
**Maddison et al. (2014)**	✓	✓		✓							✓						✓			✓					✓						
**Widmer et al. (2015)**			✓							✓	✓	✓			✓	✓	✓			✓			✓	✓		✓					

*Highlighted cells indicate that the BCTs were used in all the interventions

Objective studies with no difference between experimental and control groups

Study	1.1	1.2	1.3	1.4	1.5	1.6	1.7	1.8	2.1	2.2	2.3	2.4	2.5	2.6	2.7	3.1	3.2	3.3	4.1	5.1	6.1	6.2	7.1	8.7	9.1	10.3	10.4	11.1	11.2	12.1	12.5
Behaviour Change Technique Codes																															
Barnason et al. (2009)																								✓							
Tomita et al. (2009)											✓	✓			✓	✓	✓	✓	✓	✓					✓				✓		

Self-report studies with difference between experimental and control groups

Study	1.1	1.2	1.3	1.4	1.5	1.6	1.7	1.8	2.1	2.2	2.3	2.4	2.5	2.6	2.7	3.1	3.2	3.3	4.1	5.1	6.1	6.2	7.1	8.7	9.1	10.3	10.4	11.1	11.2	12.1	12.5
Behaviour Change Technique Codes																															
Chow et al. 2015		✓															✓			✓			✓		✓						
Frederix et al. (2015)	✓									✓							✓		✓	✓				✓				✓			
Hanssen et al. (2007)	✓																	✓	✓	✓					✓						
Reid et al. (2011)	✓									✓	✓					✓	✓		✓												

Objective studies with a difference between exp. and control groups

	Behaviour Change Technique Codes																													
**Study**	1.1	1.2	1.3	1.4	1.5	1.6	1.7	1.8	2.1	2.2	2.3	2.4	2.5	2.6	2.7	3.1	3.2	3.3	4.1	5.1	6.1	6.2	7.1	8.7	9.1	10.3	10.4	11.1	11.2	12.1	12.5
**Ammenworth et al. (2015)**	✓		✓						✓	✓	✓	✓	✓	✓	✓	✓	✓			✓											
**Antypas et al. (2014)**	✓			✓						✓						✓			✓	✓			✓								
**Devi et al. 2014**	✓				✓					✓	✓									✓				✓			✓		✓		
**Furber et al. (2010)**	✓	✓			✓					✓	✓			✓		✓															
**Reid et al. (2011)**	✓									✓	✓					✓	✓		✓												

*Highlighted cells indicate that the BCTs were used in all the interventions
